# Decidual leukocytes respond to African lineage Zika virus infection with mild anti-inflammatory changes during acute infection in rhesus macaques

**DOI:** 10.3389/fimmu.2024.1363169

**Published:** 2024-03-07

**Authors:** Michelle R. Koenig, Jessica Vazquez, Fernanda B. Leyva Jaimes, Ann M. Mitzey, Aleksandar K. Stanic, Thaddeus G. Golos

**Affiliations:** ^1^Department of Comparative Biosciences, University of Wisconsin-Madison, Madison, WI, United States; ^2^Department of Obstetrics and Gynecology, University of Wisconsin-Madison, Madison, WI, United States; ^3^Wisconsin National Primate Research Center, University of Wisconsin-Madison, Madison, WI, United States

**Keywords:** Zika virus, decidua, inflammation, immunome, rhesus macaque, pregnancy

## Abstract

Zika virus (ZIKV) can be vertically transmitted during pregnancy resulting in a range of adverse pregnancy outcomes. The decidua is commonly found to be infected by ZIKV, yet the acute immune response to infection remains understudied *in vivo*. We hypothesized that *in vivo* African-lineage ZIKV infection induces a pro-inflammatory response in the decidua. To test this hypothesis, we evaluated the decidua in pregnant rhesus macaques within the first two weeks following infection with an African-lineage ZIKV and compared our findings to gestationally aged-matched controls. Decidual leukocytes were phenotypically evaluated using spectral flow cytometry, and cytokines and chemokines were measured in tissue homogenates from the decidua, placenta, and fetal membranes. The results of this study did not support our hypothesis. Although ZIKV RNA was detected in the decidual tissue samples from all ZIKV infected dams, phenotypic changes in decidual leukocytes and differences in cytokine profiles suggest that the decidua undergoes mild anti-inflammatory changes in response to that infection. Our findings emphasize the immunological state of the gravid uterus as a relatively immune privileged site that prioritizes tolerance of the fetus over mounting a pro-inflammatory response to clear infection.

## Introduction

ZIKV was initially discovered as an infection in a sentinel macaque in Uganda in 1947 (1). Since then, ZIKV has spread from the African continent to Asia and the Pacific Islands and was introduced to Brazil between 2013 and 2014 (2, 3). It was not until after ZIKV had spread to a sizable naïve population in Brazil that a formal connection to birth defects was established (4). Severe birth defects caused by ZIKV include microcephaly and other brain abnormalities, joint contractures, ocular defects, and hearing abnormalities (5–13). Congenital infection/exposure to ZIKV can also result in fetal death, miscarriage, and intrauterine growth restriction (5, 8, 14).

ZIKV infection during pregnancy is estimated to result in birth defects or abnormal fetal development in 5–10% of infections during pregnancy (15). Maternal infection with ZIKV during the first trimester of pregnancy is associated with the greatest risk of congenital defects and other adverse outcomes (5, 8, 10). Beyond gestational timing, there are no other risk factors that have been identified to assess whether or not a pregnant person infected with ZIKV is likely to transmit the infection to their fetus (10) and there is currently no way to predict if vertical transmission will occur on an individual basis.

Maternal infection and associated inflammation, even without congenital infection, have long been associated with severe adverse pregnancy outcomes (APOs) such as preeclampsia, preterm delivery, and pregnancy loss (16, 17). Additionally, abnormal maternal inflammation during gestation is believed to interfere with normal fetal brain development resulting in neurological and behavioral problems in children (18). Although much is known about maternal systemic inflammation, how decidual inflammation and infection contribute to APOs is poorly understood (17, 19–25). The decidua is the endometrium of the pregnant uterus, mainly composed of specialized stromal cells and leukocytes. In the first trimester of human pregnancy, decidual Natural Killer (dNK) cells are the predominant leukocytes, making up ~70% of all leukocytes, followed by macrophages (~20%), T cells (~10–20%), and relatively small populations of dendritic cells (DCs) and B cells (26). Decidual stromal cells also play a crucial role in shaping the decidual immune environment, secreting cytokines that help maintain a balanced immunological environment that protects and supports the growing placenta and fetus (26). Changes in leukocyte populations in the decidua have been correlated with several APOs (22, 27). A strong pro-inflammatory response to infection mounted by decidual leukocytes could contribute to adverse pregnancy outcomes. *In vivo* and *ex vivo* studies have demonstrated that ZIKV readily infects the decidua (28–36). The acute decidual response to ZIKV infection remains essentially unstudied *in vivo*.

In our previously published study, Koenig et al. showed that African-lineage ZIKV can be vertically transmitted through the fetal membranes during early pregnancies in rhesus macaques (36). African-lineage ZIKV was used in that study because it consistently produced a high rate of vertical transmission, an ideal result for a macaque model with limited animal numbers (36). Koenig et al. showed that the decidua was consistently infected with ZIKV (36). Furthermore, infection of the decidua was shown to play an important role bridging the pathway between ZIKV infection of maternal blood and the infection the fetal membranes (36). Because the decidua is readily infected by both African and Asian ZIKV and pathological inflammation is proposed to play a role in APOs, we believe that further investigation of the effect that ZIKV has on the decidual immune environment is warranted.

We hypothesized that *in vivo* African-lineage ZIKV infection induces a pro-inflammatory response in the decidua. To test this hypothesis, we evaluated the decidua in pregnant rhesus macaques infected with an African-lineage ZIKV and compared our findings to gestationally aged-matched controls. Decidual leukocytes were phenotypically evaluated using spectral flow cytometry, and cytokines and chemokines were measured in decidual homogenates using a multiplex immunoassay. We found little evidence to support our hypothesis. Instead, we found leukocyte phenotypic changes and cytokine profiles that suggest the decidua has an anti-inflammatory response to ZIKV infection, which may aim to protect the conceptus from an inflammatory response that could compromise the pregnancy.

## Materials and methods

### Study design

This study consisted of sixteen singleton pregnancies from 15 dams; one dam served as a control twice in the study. These pregnancies fall into four distinct groups. Eight pregnant female rhesus macaques were subcutaneously inoculated with 10^4^ plaque-forming units (PFU) of a Senegal isolate of African-lineage Zika virus ZIKV/*Aedes africanus*/SEN/DAK-AR-41524/1984 (ZIKV-DAK), Genbank accession number KY348860, at approximately gestational day (gd) 30. Four of these dams had their pregnancies surgically terminated seven days post-infection (dpi), and four had their pregnancies surgically terminated at 14 dpi. These two ZIKV infected groups are referred to in this manuscript as 7-dpi-ZIKV and 14-dpi-ZIKV. Seven additional pregnant female rhesus macaques were injected with sterile saline and subject to the same experimental sampling regimen as the ZIKV-infected pregnancies. Four control pregnancies were surgically terminated seven days post-saline-injection and four at 14 days post-saline-injection. These two control groups are referred to in the manuscript as 7-dpi-control and 14-dpi-control. One of the control dams was randomly assigned to the control seven dpi group twice; all statistical analyses have been done with an average of the results from her two pregnancies. This was the only dam that contributed more than one pregnancy to this study. Details on the timing of ZIKV/saline inoculation and pregnancy termination/necropsy are provided in **Supplementary Table 1**. Details on the viral burden and pathology related to these infections can be found in the previously published manuscript (36).

### Ethics

The rhesus macaques used in this study were cared for by the staff at the Wisconsin National Primate Research Center (WNPRC) according to regulations and guidelines of the University of Wisconsin Institutional Animal Care and Use Committee, which approved this study protocol (G005691) in accordance with recommendations of the Weatherall report and according to the principles described in the National Research Council’s Guide for the Care and Use of Laboratory Animals. All animals were housed in enclosures with at least 4.3, 6.0, or 8.0 sq. ft. of floor space, measuring 30, 32, or 36 inches high, and containing a tubular PVC or stainless steel perch. Each individual enclosure was equipped with a horizontal or vertical sliding door, an automatic water lixit, and a stainless steel feed hopper. All animals were fed using a nutritional plan based on recommendations published by the National Research Council. Twice daily, macaques were fed a fixed formula of extruded dry diet (2050 Teklad Global 20% Protein Primate Diet) with adequate carbohydrate, energy, fat, fiber (10%), mineral, protein, and vitamin content. Dry diets were supplemented with fruits, vegetables, and other edible foods (e.g., nuts, cereals, seed mixtures, yogurt, peanut butter, popcorn, marshmallows, etc.) to provide variety to the diet and to inspire species-specific behaviors such as foraging. To further promote psychological well-being, animals were provided with food enrichment, human-to-monkey interaction, structural enrichment, and manipulanda. Environmental enrichment objects were selected to minimize chances of pathogen transmission from one animal to another and from animals to care staff. While in the study, all animals were evaluated by trained animal care staff at least twice daily for signs of pain, distress, and illness by observing appetite, stool quality, activity level, and physical condition. Animals exhibiting abnormal presentation for any of these clinical parameters were provided appropriate care by attending veterinarians.

### Care & use of macaques

The female macaques described in this report were co-housed with a compatible male and observed daily for menses and breeding. Pregnancy was detected by abdominal ultrasound, and gestational age was estimated as previously described (33). For physical examinations, virus inoculations, and blood collections, dams were anesthetized with an intramuscular dose of ketamine (10 mg/kg). Blood samples from the femoral or saphenous vein were obtained using a vacutainer system or needle and syringe. Pregnant macaques were monitored daily prior to and after inoculation to assess general well-being and for any clinical signs of infection (e.g., diarrhea, inappetence, inactivity, and atypical behaviors).

### Pregnancy termination and tissue collection

Pregnant dams had their pregnancies surgically terminated at gd 36–46 via laparotomy. During the laparotomy procedure, the entire conceptus (fetus, placenta, fetal membranes, umbilical cord, and amniotic fluid) was removed. The tissues for all animals were dissected using sterile instruments, which were changed between each organ/tissue to minimize possible cross-contamination. Each organ/tissue was evaluated grossly *in situ*, removed with sterile instruments, placed in a sterile culture dish, and dissected. Tissue samples collected for cytokine and chemokine analysis were flash-frozen on dry ice and stored at -80 °C. The decidua basalis was removed from the placenta and temporarily stored in sterile PBS on ice.

### Single-cell isolation of decidual basalis leukocytes and PBMCs

Freshly collected decidua basalis was washed with cold PBS and processed using methods previously described (37). Briefly, tissue was minced and dissociated in RPMI containing 1 mg/mL of Collagenase from *Clostridium histolyticum* (Sigma Aldrich, St. Louis, MO) and 1 μg/mL Deoxyribonuclease I from bovine pancreas (Sigma Aldrich, St. Louis, MO), using the GentleMACS Dissociator system (Miltenyi Biotec Inc., San Diego, CA, USA). Homogenates were then filtered through a 100-μm filter; red blood cells were lysed with ACK Lysis Buffer (Lonza, Walkersville, MD), and mononuclear cells (MCs) were recovered and frozen in CryoStor CS5 (BioLife Solutions, Bothell, WA) and stored in liquid nitrogen before evaluation. PBMCs were isolated from peripheral blood collected in EDTA tubes using previously described standard methods (38), frozen in CryoStor CS5, and stored in liquid nitrogen prior to evaluation. PBMC samples taken on the day of pregnancy termination were used for most cases. If such a sample was not available, the closest available PBMC sample, prior to surgical pregnancy termination, was used. Full details on the PBMC samples used can be found in **Supplementary Table 1**.

### Flow cytometry

Isolated MC suspensions from the decidua and peripheral blood were removed from liquid nitrogen, thawed, and washed. All decidua and PBMC samples were thawed, stained, and evaluated in one batch to avoid batch effects. After washing, MCs were labeled with the viability marker Zombie NIR (Biolegend, San Diego, CA) according to the manufacturer’s instructions. MCs were then labeled with surface marker fluorochrome-conjugated monoclonal antibodies (**Table 1**, **Supplementary Table 2**). Briefly, antibodies, True-Stain Monocyte Blocker, and TrueStain FcX Block (BioLegend, San Diego, CA) were diluted in BD Horizon Brilliant Stain Buffer (BD Biosciences, San Jose, CA, USA) and used to label MCs. Following a 30-minute incubation at room temperature, samples were washed and treated with FoxP3/Transcription factor staining buffer set (Invitrogen a Life Technologies Corporation, Carlsbad, CA) to permeabilize and fix cells overnight (approximately 13 hours) at 4°C. Samples were washed and stained with intracellular fluorochrome-conjugated monoclonal antibodies (**Table 1**). Intracellular antibodies were added sequentially, mixing each time by pipetting up and down, in the following order: T-bet, RORγt, FoxP3, Eomes. Each intracellular antibody was incubated for 15 minutes before adding the next antibody, and samples were incubated for 30 additional minutes after the last antibody (Eomes) was added. After incubation with intracellular antibodies, samples were washed, resuspended, and filtered into Falcon 5mL polystyrene round-bottom tubes with cell-strainer caps (Corning, Corning, NY). In addition, unstained controls, fluorescent minus-multiple gating controls, and single stained controls using decidual MCs, PBMCs, and UltraComp eBeads compensation beads (Invitrogen a Life Technologies Corporation, Eugene, OR) were also prepared. Samples were then acquired using the 5-laser Cytek Aurora Spectral flow cytometer (Cytek Biosciences, Fremont, CA). Raw data were exported as FCS files.

**Table 1:** Antibodies used for flow cytometry.

Marker	Clone	Fluorochrome	Supplier
CD45	D058-1283	BUV395	BD Biosciences
CCR6	11A9	BUV496	BD Biosciences
CD45RA	5H9	BUV563	BD Biosciences
CD4	SK3	BUV615	BD Biosciences
CD69	FN50	BUV661	BD Biosciences
CD8α	SK1	BUV805	BD Biosciences
FoxP3	206D	BV421	BioLegend
CD16	3G8	BV480	BD Biosciences
CD14	M5E2	BV570	BioLegend
CD49a	SR84	BV605	BD Biosciences
CD163	GHI/61	BV650	BD Biosciences
CD3	SP34-2	BV711	BD Biosciences
CCR7	G043H7	BV785	BioLegend
CD56	B159	BB515	BD Horizon
CD20	2H7	PerCP Cy5.5	BioLegend
RORγ-t	AFKJ5-9	PE	Invitrogen
CD11c	3.9	PE/Dazzle 594	BioLegend
T-bet	ebio4B10	PE-Cy5	Invitrogen
CD127	A019D5	PE/Fire700	BioLegend
NKp46	–	PE-Cy7	Beckman Coulter
CD86	IT2.2	PE/Fire810	BioLegend
*DC-SIGN	DCN46	APC	BD Biosciences
Eomes	WD1928	eFluor 660	Invitrogen
Viability	NA	Zombie NIR	BioLegend

*DC-SIGN antibody was included in the staining cocktail but was excluded from analysis due to poor signal.

### Spectral unmixing

**Figure 1:**
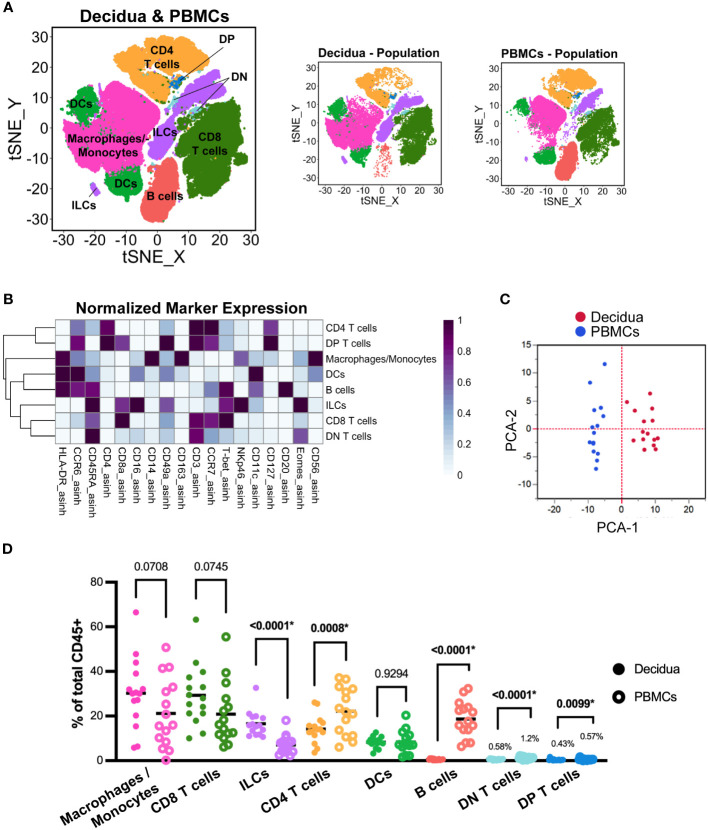
Evaluation of total leukocytes in decidual and PBMC samples from all pregnancies. **(A)** t-SNE map generated from pre-gated (live, CD45+, single cells) leukocytes from the decidua and PBMCs showing CD4 T cells, CD8 T cells, macrophage/monocytes, dendritic cells (DC), innate lymphoid cells (ILCs), B cells, CD8+ CD4+ double-positive (DP) T cells, and CD8- CD4- double-negative (DN) T cells identified by FlowSOM clustering. Panels to the right show the distribution of those cells, specifically within decidua and PBMC samples. **(B)** Heatmap of each marker’s median fluorescent intensity (MFI) in the clusters identified by FlowSOM. Marker expression is normalized by column. **(C)** Principal component analysis of the MFI of each marker within each cluster as shown in **(B)** from all decidua (n=15) and PBMC (n=15) samples. **(D)** Scatter plot of the frequency of the major immune cell populations found in each decidual (n=15) and PBMC (n=15) sample. The relative frequency of each population was compared using a paired t-test; the p values for each test are shown; * indicates significant p values (p < 0.05 is considered significant).

Processing of raw data was done with SpectroFlo (Cytek Biosciences, Fremont, CA) to unmix the fluorescent signals acquired by the Cytek Aurora spectral flow cytometer (Cytek Biosciences, Fremont, CA). Following previously published guidelines on best practices, PBMC and decidua samples were unmixed separately using a respective set of optimized controls (39). The control samples were pre-gated to improve reproducibility. Autofluorescence was extracted during the unmixing process using a matched unstained sample. The samples used to create the principal component analysis (PCA) plot in **Figure 1** were all unmixed using the decidua unmixing template. The PBMCs samples unmixed in this manner were exclusively used to create this PCA plot. All other analysis of the PBMCs was done on samples unmixed using their own set of single stain controls. Unmixed samples were exported as FSC files. To avoid any potential cofounders due to different unmixing methods and autofluorecent extraction, the median fluorescent intensity (MFI) was not compared between PBMCs and decidua.

### Flow cytometry data analysis

Unmixed FSC files were pre-processed in FlowJo v.10.8 software (FlowJo LLC, Ashland, OR, USA) to remove debris, doublets, non-leukocytes (CD45-), and dead cells (**Supplementary Figure 1**), resulting in a “total leukocytes” population for each sample. Samples were then downsampled to 50,000 cells using the FlowJo plugin Downsample (V3.2). FCS files were then exported as CSV - scale value files from FlowJo.

### Dimensionality reduction and cluster identification

Pre-processed PBMC and decidua flow cytometry data were analyzed using the Specter R package (40), a comprehensive toolbox that allows for transformation, clustering, annotation, and visualization (tSNE plots, heatmaps) of high-dimensional flow cytometry data. All R scripts used for data analysis have been deposited on GitHub at https://github.com/Team-Placenta-Golos-Lab/ZIKV-DAK-decidua-immune. All flow data were arcsine transformed and cofactor values were determined for each marker selected based on the location of positive and negative populations, informed by fluorescent minus-multiple control samples. FlowSOM (41) clustering and t-distributed stochastic neighbor embedding (t-SNE) visualization (42) were implemented through Specter. To compare the relative frequencies of major leukocyte populations, PBMCs and decidua samples were analyzed together. This process was repeated on the decidua samples alone.

We first performed an “overview’ level clustering analysis. This allowed us to identify the major leukocyte populations within the decidua. We performed additional clustering analysis to effectively “zoom in” on the overview clusters (e.g., T cells) to further evaluate each major decidual leukocyte population. This approach avoids artifacts introduced by the unavoidable spillover of markers; additionally, it allows for the customization of arcsine transformations to optimize the visualization of markers that may be expressed differently in, for example, T cells vs. macrophages.

### Statistical analysis of flow cytometry data

One dam was randomly assigned to the 7-dpi-control group twice; thus two of the four control 7 dpi pregnancies are from the same dam. All statistical analyses were done using averaged data from this dam’s two pregnancies (averaged decidua data and PBMC data). Thus, in all graphs depicting data from both ZIKV infected and control pregnancies there is a total n=15, since for controls n=7. Statistical analyses that directly compared PBMCs and decidua used paired t-tests. Comparisons between groups (7-dpi-ZIKV, 14-dpi-ZIKV, 7-dpi-control, 14-dpi-control) were made using a one-way ANOVA. If the one-way ANOVA was significant, a Bonferroni comparison test was done comparing the following pairs: 7-dpi-control vs. 7-dpi-ZIKV, 7-dpi-control vs. 14-dpi-control, 14-dpi-control vs. 14-dpi-ZIKV, and 7-dpi-ZIKV vs 14-dpi-ZIKV. Comparisons between treatment groups (ZIKV vs. controls) were done using results from pooled ZIKV samples (7-dpi-ZIKV and 14-dpi-ZIKV) and pooled control samples (7-dpi-Control and 14-dpi-Control) using a two-tailed t-test. P values below 0.05 were considered significant. All statistical analysis was done with Prism v. 9 (GraphPad Software Inc, La Jolla, CA, USA) or JMP Pro v. 15 (SAS, Cary, NC, USA). PCA plots were created using JMP Pro v. 15.

### Tissue homogenization and protein quantification

Flash-frozen tissues were thawed and homogenized using standard methods. Briefly, tissue biopsies were cut into small pieces and placed in a snap cap tube with PBS, 10 μl/mL of Halt Protease and Phosphatase Inhibitor Cocktail 100X (Thermo Scientific, Waltham, MA), and 3.2 mm stainless-steel beads. Tubes were then homogenized with a TissueLyser II (Qiagen, Germantown, Maryland) run at 30 frequency for 2 minutes, three times. Samples were then centrifuged to remove any debris. The resulting supernatant was analyzed for protein concentration using the Micro BCA Protein Assay and read on the SpectraMax Plus 384 Microplate Reader (Molecular Devices). The samples were then frozen and stored at -80°C until assayed.

### Cytokine and chemokine analysis

Samples were analyzed using the LEGENDplex NHP Inflammation Panel (13-plex) with V-bottom Plate (Biolegend, San Diego, CA) multiplex assay according to the manufacturer’s protocol. Tissue homogenates were thawed and diluted to reach a standard concentration to ensure each sample had an equal amount of protein evaluated. Input protein concentration was standardized to 1.26 μg for decidual and chorionic villous homogenates and 1.09 μg for chorionic plate, chorionic membrane, and amniotic membranes. All samples were run in duplicate. The assay beads were fixed with 4% paraformaldehyde (PFA) for five minutes. Samples were then analyzed on the Attune NxT Flow Cytometer (ThermoFisher Scientific, Waltham, MA). The resulting raw FCS files were analyzed using LEGENDplex Data Analysis Software Suite (Biolegend, San Diego, CA). IFN-γ results were excluded from the first plate that contained the decidua samples because a standard curve could not be calculated from the standards. The mean value was calculated from each duplicate well. The raw LEGENDplex assay data is available at http://flowrepository.org/id/FR-FCM-Z6VZ and http://flowrepository.org/id/FR-FCM-Z6V3.

### Statistical analysis of cytokine and chemokine data

Comparisons between treatment groups (ZIKV vs. controls) were made using a two-tailed t-test, and comparisons between groups (7-dpi-ZIKV, 14-dpi-ZIKV, 7-dpi-control, 14-dpi-control) were made using a one-way ANOVA. If the one-way ANOVA was significant, a Bonferroni comparison test was done comparing the following pairs: 7-dpi-control vs. 7-dpi-ZIKV, 7-dpi-control vs. 14-dpi-control, 14-dpi-control vs. 14-dpi-ZIKV, and 7-dpi-ZIKV vs 14-dpi-ZIKV. For the chorionic membrane and amniotic membrane samples, 7-dpi-Control samples were compared to 7-dpi-ZIKV samples using a two-tailed t-test. P values below 0.05 were considered significant. All statistical analysis was done with Prism v. 9 or JMP Pro v. 15. PCA plots were created using JMP Pro v. 15. Graphs were created using Prism v.9.

## Results

### Decidual immune cells are distinct from those present in the peripheral blood

As shown in Koenig et al., all of the eight dams challenged with ZIKV-DAK became productively infected, with plasma viral loads peaking between 4 and 7 dpi (36). ZIKV was detected in the decidua in all eight pregnancies at 7 dpi, and at 14 dpi. The viral load was increased in the decidua at 14 dpi compared to 7 dpi (36). Cellular localization of ZIKV in the decidua suggested the majority of the infection in the decidua was within extravillous trophoblasts and a small number of decidual macrophages were also found to be infected (36). To better understand the decidual immune response to ZIKV infection, we collected the decidua either at 7 or 14 dpi from eight ZIKV infected dams and from eight mock infected dams (**Supplementary Figure 2**). Major immune cell subsets were assessed simultaneously across all treatment groups using a highly polychromatic spectral flow cytometry panel.

We first wanted to determine the similarities between decidual and peripheral leukocytes from all pregnancies. FlowSOM clustering coupled with t-SNE visualization identified major leukocyte populations: CD8+ Cytotoxic T cells, CD4+ T helper cells, CD4- CD8- (double negative, DN) T cells, CD4+ CD8+ (double positive, DP) T cells, macrophages/monocytes, DCs, innate lymphoid cells (ILCs), and B cells (**Figure 1A**). We based our annotations on the expression of lineage-defining markers (**Table 2**, **Figure 1B**) following previously reported phenotypes (37, 43–45). To summarize the differences between the PBMCs and the decidua, PCA was performed on the MFI of each marker in each of the eight major leukocyte populations on identically unmixed sample files. The resulting PCA plot clearly separates decidua and PBMC populations (**Figure 1C**), suggesting that decidual and PBMC leukocyte populations are distinct. To confirm this observation, we compared the relative frequency of major leukocyte populations between the paired PBMC and decidua samples from all pregnancies (**Figure 1D**). This analysis found that the frequency of ILCs was significantly higher (p <.0001) in the decidua, while the frequency of CD4+ T cells (p = 0.0008), B cells (p <.0001), DN (CD4- CD8-) T cells (p <.0001), and DP (CD4+ CD8+) T cells (p = 0.0099) was significantly lower compared to the PBMCs (**Figure 1D**). Although not statistically significant, there was a trend towards a higher proportion of macrophages and CD8 T cells in the decidua than the PBMCs (p = 0.0708, 0.0745, respectively). Collectively, these data suggest that decidual leukocytes evaluated in this study represent a population distinct from PBMCs.

**Table 2:** Defining marker expression of major leukocyte populations.

Leukocyte population name	Defining marker expression
CD8+ Cytotoxic T cells	CD45+ CD3+ CD8+ CD4-
CD4+ helper T cells	CD45+ CD3+ CD8- CD4+
CD4- CD8- T cells (Double negative T cells)	CD45+ CD3+ CD8- CD4-
CD4+ CD8+ T cells (Double positive T cells)	CD45+ CD3+ CD8+ CD4+
Macrophages/Monocytes	CD45+ CD3- CD14+ HLA-DR+
Dendritic cells (DCs)	CD45+ CD3- CD14- CD163- HLA-DR^high^
Innate lymphocyte cells (ILCs)	CD45+ CD3- CD14- CD163- HLA-DR^low/-^ CD20^low/-^
B cells	CD45+ CD3- CD20^mid/high^

### Leukocyte composition of the rhesus macaque decidua at early gestation more closely resembles that found in later gestation in humans

**Figure 2:**
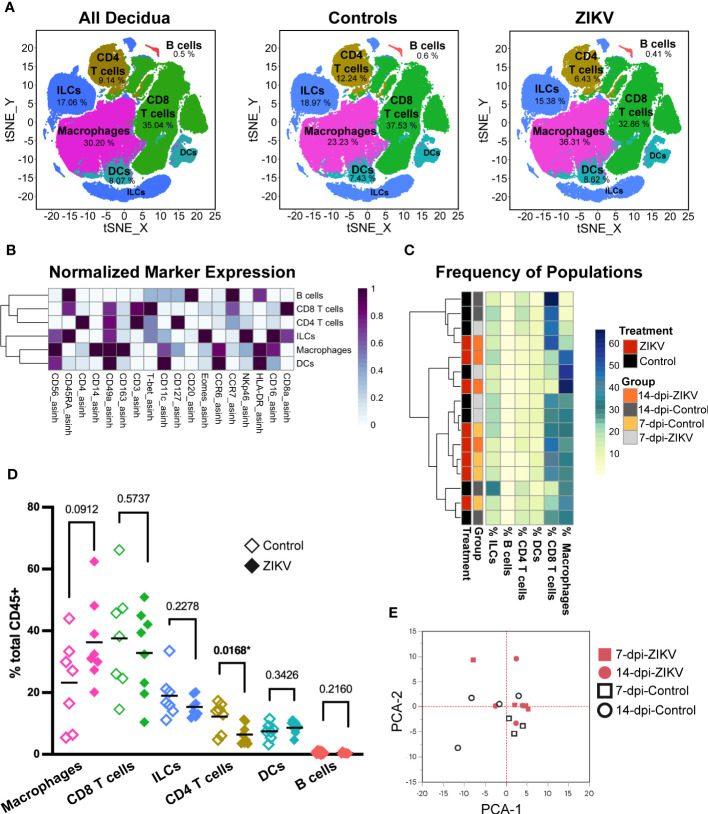
Evaluation of total leukocytes in decidua samples from ZIKV- and control-treated dams. **(A)** t-SNE map generated from pre-gated leukocytes from the decidua showing CD4 T cells, CD8 T cells, macrophages, dendritic cells (DC), innate lymphoid cells (ILCs), and B cells identified by FlowSOM clustering. Left panel: all samples, center: control samples, right: ZIKV samples. The respective percentage for each population is shown under each population name. **(B)** Heatmap of the MFI of each marker in the clusters identified by FlowSOM. Marker expression is normalized by column. **(C)** Heatmap of leukocyte population frequencies. Each row represents one sample. Each sample’s treatment and group identity is annotated on the left side of the heatmap. **(D)** Scatter plot of the frequency of the major immune cell populations found in pooled ZIKV vs. Control samples. The relative frequency of each population was compared using a t-test; the p values for each test are shown; * indicates significant p values. **(E)** Principal component analysis of the MFI of each marker and cluster as shown in **(B)**.

In humans, the composition of decidual leukocytes changes throughout gestation. In the first trimester of pregnancy, decidual natural killer cells (dNKs) account for ~70% of all decidual leukocytes (46). As gestation continues, the frequency of T cells increases, and the frequency of dNKs decreases, and eventually, T cells become the predominant decidual leukocyte (47). Natural killer (NK) cells are a type of innate lymphocyte cell (ILC) and we have classified dNKs as ILCs to acknowledge the diversity of dNKs and the presence of other ILCs in the decidua (45, 48–50). Decidual cells were evaluated alone with FlowSOM and tSNE visualized using tSNE (**Figure 1A**) to identify major leukocyte populations based on the expression of lineage-defining markers (**Figure 2B**). Evaluation of the pooled control decidual samples alone shows that CD8+ T cells are the most abundant leukocytes (mean ± SD, 37.53 ± 17.36%) found in the decidua, followed by macrophages (23.23 ± 14.38%), ILCs including dNKs (18.97 ± 7.28%), CD4+ T cells (12.24 ± 4.91%), DCs (7.43 ± 2.64%), and B cells (0.6 ± 0.28%) (**Figures 2A, C, D**). These results differ from those previously reported in rhesus macaque decidua at around the same gestational age, where dNKs were found to account for ~70% of all leukocytes (43, 51). However, the current findings more closely resemble those recently reported by Moström et al., who also found CD8+ T cells make up the highest percentage of decidual leukocytes in rhesus macaques (44). This distribution of decidual leukocytes found in our control samples suggests that the composition of rhesus macaque decidual leukocytes at this time in gestation more closely resembles the decidual immune population seen in humans later in gestation (47, 52, 53).

### ZIKV infection has minimal impact on the relative frequency of major decidual leukocyte populations

The clustering of major decidual leukocyte populations created an overview level of analysis. Hierarchical clustering of the frequencies of each leukocyte population within the sample revealed no overall pattern between ZIKV and control decidua samples (**Figure 2C**). The frequency of each population was compared between pooled ZIKV and control samples (**Figure 2D**). We found that ZIKV-exposed decidua samples had a significant decrease in the frequency of CD4 T cells (p = 0.0168). ZIKV-exposed decidual samples also had an increase in macrophages, but these findings were not statistically significant (p = 0.0912) (**Figure 2D**). PCA analysis of ZIKV and control decidua samples shows that although some samples clustered together, there is no clear separation of ZIKV vs. Control groups (**Figure 2E**). Decidual DCs and B cells were successfully identified. ZIKV had no detectable effect on these populations. Due to the limited number of cells and the lack of appropriate markers for further phenotyping, no further analysis was done on these populations. Macrophages, T cell, and ILCs were further evaluated to determine if ZIKV causes more subtle changes in these decidual leukocytes.

### ZIKV infection correlates with an increase in scavenger functions of decidual macrophages

**Figure 3:**
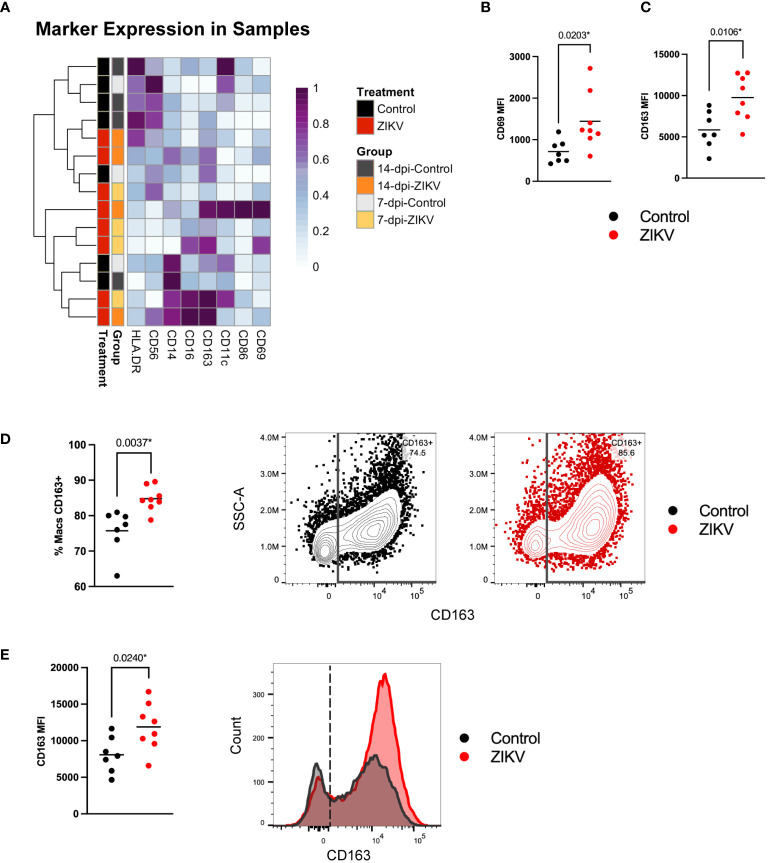
Decidual macrophage populations in ZIKV and control samples. **(A)** Heatmap of the MFI of each macrophage marker. Marker expression is normalized by column. Each row represents one sample for which the group and treatment are annotated on the left side. **(B)** Scatter plots showing the MFI of CD69 and **(C)** the MFI of CD163 in macrophages from ZIKV and control samples. **(D)** Scatter plot showing the frequency of CD163+ macrophages within all macrophages for each sample. Flow plots to the right show gating of CD163+ macrophages in representative control (black) and ZIKV (red) samples. **(E)** Scatter plot showing the MFI of CD163 in CD163+ macrophages and a histogram of the data shown in the flow plots in **(C)**. Comparisons were made using a t-test; * indicates a statistically significant p value.

We assessed the diversity of macrophages and analyzed marker expression across our samples (**Figure 3A**). Most of the decidual macrophages expressed CD163, CD56, CD11c, and CD86; a minority expressed CD16 and CD69 (**Supplementary Table 3**). These findings are consistent with previous studies (26, 44, 54, 55). We further compared the MFI of each macrophage marker between ZIKV and control samples to assess any potential functional differences. We found a significant increase in CD69 expression in ZIKV samples (**Figure 3B**). CD69 is an activation marker that is induced in murine macrophages stimulated with IFN-γ and bacterial lipopolysaccharides (LPS) (56). There was also a significant increase in CD163 expression in macrophages from ZIKV samples (**Figure 3C**). Further analysis showed that ZIKV-exposed decidua had a higher frequency of CD163+ macrophages (**Figure 3D**), and even within the CD163+ macrophages, CD163 was expressed at higher levels (**Figure 3E**). CD163+ macrophages are believed to play a role in controlling inflammation by scavenging components of damaged cells (57). Overall, the differences found suggest that decidual macrophages respond to ZIKV infection primarily by increasing their scavenging functions, a change that is consistent with an anti-inflammatory response (57).

### ZIKV infection alters transcription factor expression in select ILCs

The recent redefining of NK cells as part of a larger group of innate lymphoid cells adds additional complexity to our understanding of immune responses in the decidua (37, 45, 58, 59). ILCs have been extensively studied in the human decidua (45, 60–64). Decidual NKs are believed to play an essential role in spiral artery remodeling (61, 65, 66) and have been implicated in a number of pregnancy pathologies (67, 68). Three basic types of ILCs have been identified and are distinguished by different transcription factors: ILC1 cells, which include NK cells, express T-bet; ILC2 cells express GATA-3; and ILC3 cells express RORγt. The majority of ILCs in human decidua are CD56^bright^ CD16- NK cells, which have been further distinguished by their expression of Eomes (45, 48). A rhesus counterpart of CD56^bright^ CD16^dim^ dNK has also been identified (43, 51). In the current study, decidual ILCs were identified as lineage negative (lin-) (CD3-, CD14-, HLA-DR^low/-^, CD20-) (**Table 2**). Consistent with previously published studies, most rhesus decidual ILCs are CD56+ CD16-/low (61.49 ± 12.34%) (**Supplementary Figure 3A**). In comparing all decidual ILCs from ZIKV samples to controls, we found a significant increase in the expression of Eomes (p = 0.0026) (**Supplementary Figure 3B**).

**Figure 4:**
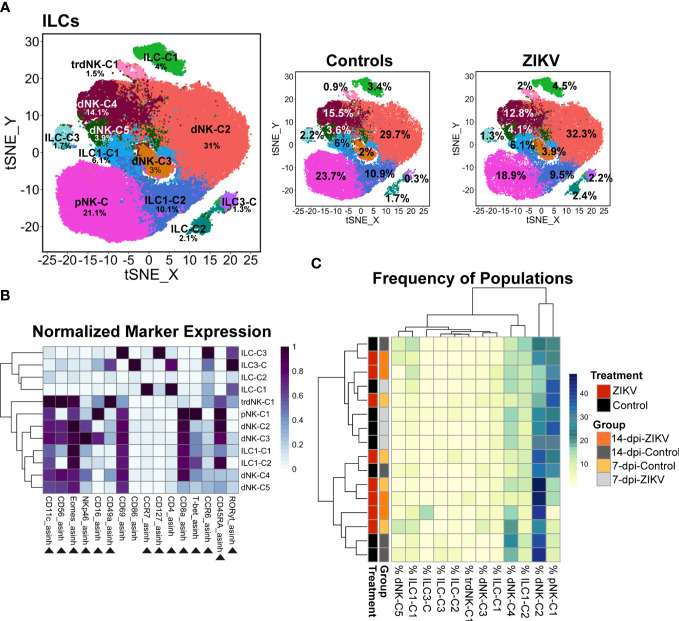
Evaluation of decidual ILCs from ZIKV and control samples. **(A)** t-SNE map generated from pre-gated ILCs from the decidua showing the populations identified by FlowSOM clustering. The left panel shows decidual ILCs from all samples, the center panel control, and the right panel ZIKV. The respective percentage for each population is shown under each population name. **(B)** Heatmap of the MFI of each marker in the clusters identified by FlowSOM. Marker expression is normalized by column. Black triangles indicate markers used for clustering. **(C)** Heatmap of the ILC population frequencies. Each row represents one sample. Each sample’s treatment and group identity is annotated on the left side of the heatmap.

To further evaluate ILCs, we performed additional clustering analysis using relevant markers. Using this “zoom in” method, we increased the granularity of our analysis and identified 12 ILC cluster subsets (**Figures 4A, B**). Clusters were annotated further to reflect their major phenotype. Classical dNKs express CD56, and in the rhesus macaque are CD16- or express low levels of CD16. The majority of the decidual ILCs were CD56+ CD16 low/- a phenotype that is consistent with classically described dNKs; these dNKs are annotated as the following clusters: tissue-resident (tr)dNK-C1, dNK-C2, dNK-C3, dNK-C4, and dNK-C5. In addition to being CD56+ CD16^low/-^, these clusters also express the transcription factor Eomes. The trdNK-C1 cluster uniquely expressed a high level of CD49a (**Figure 4B**), a marker of tissue residency (69, 70). One cluster, designated as pNK, was CD56- CD16+ CD8+ T-bet+, closely resembling the phenotype seen in the peripheral NK cells. Additional clusters of ILCs were identified (**Figure 4B**). Two identified clusters that were CD56- CD8+ Eomes+ T-bet- appear to resemble ILC1s. Although these cells lack T-bet expression, previously published studies have suggested that tissue ILCs don’t require T-bet expression to be considered an ILC1 as long as either T-bet or Eomes is expressed (71–74). Therefore, these clusters have been identified as ILC1-C1 and ILC1-C2 because they resemble ILC1s. One cluster was found to express RORγt, the transcription factor that defines ILC3s and is thus called ILC3-C. A small percentage of ILCs were clustered into the remaining three clusters. These clusters lacked the rhesus macaque NK cell markers CD8 and CD56, and two of these clusters do not express transcription factors Eomes, T-bet, and RORγt; thus, these clusters are designated ILC-C1, ILC-C2, and ILC-C3.

**Figure 5:**
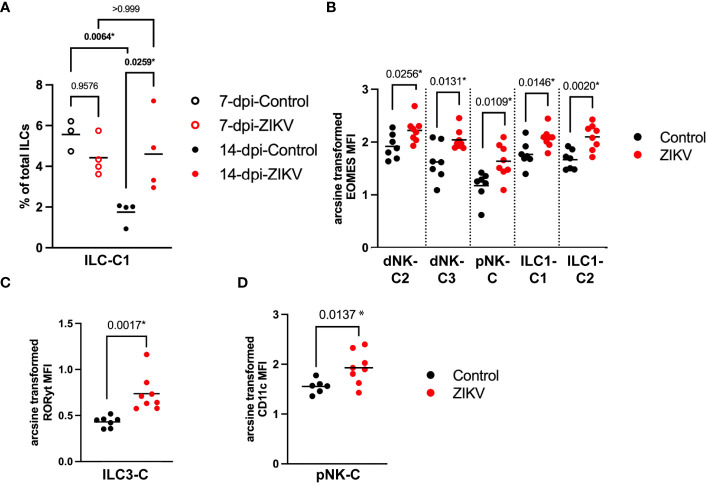
Comparison of decidual ILCs between ZIKV and control samples. **(A)** Scatter plot showing the percent of ILC-C1 at the group level. Significance was determined by a one-way ANOVA followed by a Bonferroni’s comparison test. **(B)** Scatter plot comparing the arcsine transformed MFI of Eomes per sample in the four clusters labeled on the x-axis. **(C)** Comparison of the MFI of RORγt in ILC3-C of ZIKV and control samples. **(D)** Scatter plot showing the arcsine transformed MFI of CD11c in control samples. **(B-D)** Comparisons were made using a t-test; * indicates a statistically significant p value.

In comparing the frequency of the 12 different decidual ILC clusters, there were no significant differences in the frequency of the clusters between all ZIKV and all control samples, nor was any particular pattern revealed by hierarchical clustering (**Figure 4C**). There was a significant difference at the group level in the frequency of ILC-C1 (ANOVA p = 0.0072) with a significant increase of this cluster in the 14-dpi-ZIKV samples when compared to the 14-dpi-Control samples (Bonferroni p = 0.0259) (**Figure 5A**).

Eomes is a transcription factor that regulates IFNγ expression and has been shown to play a role in NK cell development and maturation in mice (75, 76). Eomes was increased in dNK-C2, dNK-C3, and pNK-C in ZIKV vs. control samples (**Figure 5B**). Eomes expression was also significantly increased in both ILC1-like clusters (**Figure 5B**). The MFI of RORγt, a regulator of pro-inflammatory cytokines IL-17 and IL-22 (77, 78), was significantly increased in ILC3-C of ZIKV decidua samples (**Figure 5C**).

To further evaluate the potential effect of ZIKV on decidual ILCs we evaluated the expression of markers expected to increase during a pro-inflammatory response. We found that the expression of the activation marker CD69 was not significantly different between ZIKV and control samples in any of the ILC clusters. CD11c, a complement receptor that is associated with pro-inflammatory functions in mice (79, 80), was found to be increased in pNK-C in ZIKV samples (**Figure 5D**, **4B**). However, overall, there were no clear phenotypic changes in the ILCs consistent with a pro-inflammatory response.

### Cytotoxic T cells

**Figure 6:**
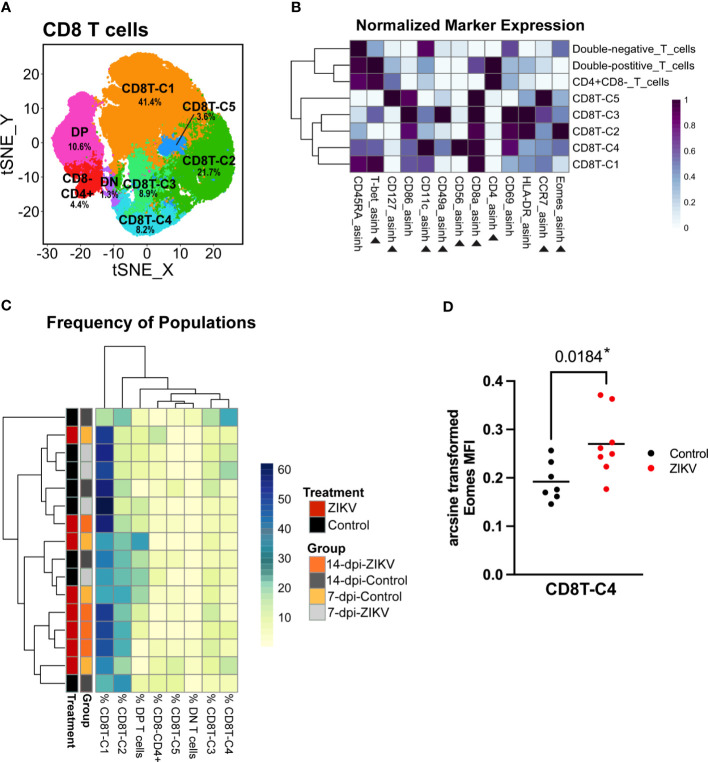
Evaluation of decidual Cytotoxic T cells from ZIKV and control samples. **(A)** t-SNE map generated from pre-clustered CD8+ Cytotoxic T cells from the decidua showing populations identified by FlowSOM clustering. DN, double-negative T cells (CD8- CD4-); DP, Double-positive T cells (CD8+ CD4+). **(B)** Heatmap of the MFI of each marker in the clusters identified by FlowSOM. Marker expression is normalized by column. Black triangles indicate markers used for clustering. **(C)** Heatmap of the CD8 T cell population frequencies. Each row represents one sample. Treatment and group identity of each sample is annotated on the left side of the heatmap. **(D)** A scatter plot comparing the arcsine transformed MFI of Eomes in ZIKV and control samples in CD8T-C4. Comparisons were made using a t-test; * indicates a significant p value.

CD8 T cells comprised a large percentage of the total decidual leukocytes for both control and ZIKV samples. Overall, no meaningful differences were found between decidual CD8 T cells in ZIKV vs. control samples. To further evaluate the decidual CD8 T cells, we performed an additional clustering analysis using relevant markers (**Figure 6**). This analysis revealed five CD8+ CD4- cytotoxic T cell populations (CD8T-C1–C4), a DP (CD8+ CD4+) population, a CD8- CD4+ population, and a DN (CD8- CD4-) population (**Figures 6A, B**).

There were no significant differences in the frequency of any of the five CD8 T cell populations between ZIKV and controls; however, the hierarchical clustering did show some grouping of ZIKV samples together (**Figure 6C**). The majority of decidual CD8 T cells were clustered into CD8T-C1 (41.36 ± 12.105%) that express high levels of T-bet (**Figure 6B**). The only significant finding related to potential pro-inflammatory changes was the increase in the expression of Eomes in select CD8 T cell populations. Eomes was significantly increased in the CD8T-C4 subset, which uniquely expresses CD56 (**Figures 6B, D**); CD8+ CD56+ T cells have been previously reported and studied in the human decidua (81). CD56 expression has been shown to allow T cells to obtain innate-like properties, allowing for T cell receptor (TCR)-independent activation through antigen-nonspecific signals and cytokines (82–86). CD56 expression has also been shown to restrict cytokine responses in T cells (87). However, it should be noted that the functions of these CD56+ T cells or any other leukocytes were not evaluated in the current study.

To compare the CD8 T cells found in the decidua to previously published studies, CD8+ T cells were gated in FlowJo based on CD45RA and CCR7 expression to identify memory subsets. Using CD45RA and CCR7 expression, 35.71 ± 18.66% of decidual CD8+ T cells were identified as effector memory (CD8-T_EM_), 47.64 17.68% as effector (CD8-T_E_), 7.69 ± 3.50% as central memory (CD8-T_CM_) and 8.96 ± 5.39% as naive (CD8-T_N_) (**Supplementary Figure 4A**). Compared to previously published studies, our results show an unexpectedly high percentage of CD8-T_E_ and CD8-T_N_ in both the decidua and the PBMCs (37, 44, 88–90). This difference remains even when we look at control samples only. We also evaluated the memory subsets of our CD4+ T cells. We found that 23.14 ± 7.50% of decidual CD4 T cells were identified as effector memory (CD4-T_EM_), 55.29 ± 10.98% as effector (CD4-T_E_), 4.78 ± 2.20% as central memory (CD4-T_CM_) 12.11 ± 7.31% as naive (CD4-T_N_) (**Supplementary Figure 4B**). Consistent with the CD8 T cell results, we found more CD4 T effector cells and fewer naive cells compared to previously published studies (91).

### T helper cells

**Figure 7:**
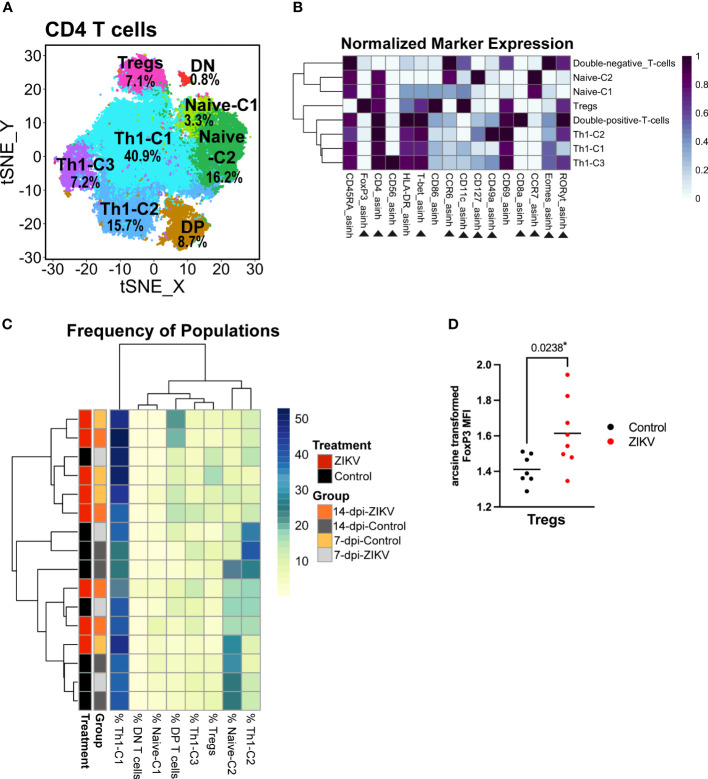
Evaluation of decidual helper T cells from ZIKV and control samples. **(A)** t-SNE map generated from pre-gated CD4+ helper T cells from the decidua showing populations identified by FlowSOM clustering. DN = double-negative T cells (CD8- CD4-). DP = Double-positive T cells (CD8+ CD4+). **(B)** Heatmap of the MFI of each marker in the clusters identified by FlowSOM. Marker expression is normalized by column. Black triangles indicate markers used for clustering. **(C)** Heatmap of the CD4 T cell population frequencies. Each row represents one sample. Treatment and group identity of each sample is annotated on the left side of the heatmap. **(D)** Scatter plot comparing the arcsine transformed MFI of FoxP3 in ZIKV and control cells in the Treg cluster. Comparisons were made using a t-test; * indicates a significant p value.

As previously discussed, there was an overall decrease in the frequency of CD4+ T helper cells in ZIKV decidua samples compared to controls (**Figure 2D**). However, no other effect on global decidual CD4 T cells was found. We then zoomed in on the CD4 T cell cluster and performed additional clustering analysis using relevant markers. This analysis revealed six CD4+CD8- populations, a small CD4+ CD8+ population (DP-T-cells) and a small CD4-CD8- population (DN-T-cells) (**Figure 7A**). Similar to ILCs, CD4+ T helper cell subsets are identifiable by the expression of transcription factors: Th1 T helper cells express T-bet, Th22 cells express Gata-3 (not included in this panel), Th17 and Th22 cells express RORγt, and T-regulatory cells (T_Regs_) express FoxP3. Three T-bet-expressing clusters were identified and annotated as Th1-C1, Th1-C2, and Th1-C3, as these clusters have phenotypes consistent with Th1 cells (**Figure 7B**). A cluster of T_Regs_ was identified by its expression of FoxP3 and relatively low expression of CD127 (**Figure 7B**). The remaining two clusters express high levels of CCR7 and CD45RA, suggesting that these are naive CD4 cells and thus are annotated as Naive-C1 and Naive-C2 (**Figure 7B**).

Although globally, ZIKV-infected samples had a decrease in the frequency of CD4 T cells, this decrease was not seen within any specific CD4 T cell population. The only difference in the relative frequency was an increase of the double-positive-T-cell cluster in the ZIKV samples vs. the controls (p = 0.0309) (**Supplementary Figure 5**). Hierarchical clustering of the frequency of the identified CD4 T cell populations in each sample did not reveal any particular pattern (**Figure 7C**). The only meaningful and significant finding in the CD4+ T helper cells was the increase in FoxP3 expression in the Treg cluster in the ZIKV samples compared to the controls (**Figure 7D**). Collectively, although a decrease in CD4 T cells has been shown to correlate with systemic inflammation, we found no phenotypical changes indicative of a pro-inflammatory response.

### Cytokine profiles of decidual homogenates do not support a pro-inflammatory response in the decidua

To further evaluate the decidual immune environment, cytokines were evaluated in decidual tissue homogenates using the LEGENDplex NHP Inflammation Panel multiplex assay. Cytokines and chemokines were also assessed in tissues homogenates from the chorionic villi of the placenta, chorionic plate of the placenta, chorionic membrane, and amniotic membrane.

**Figure 8:**
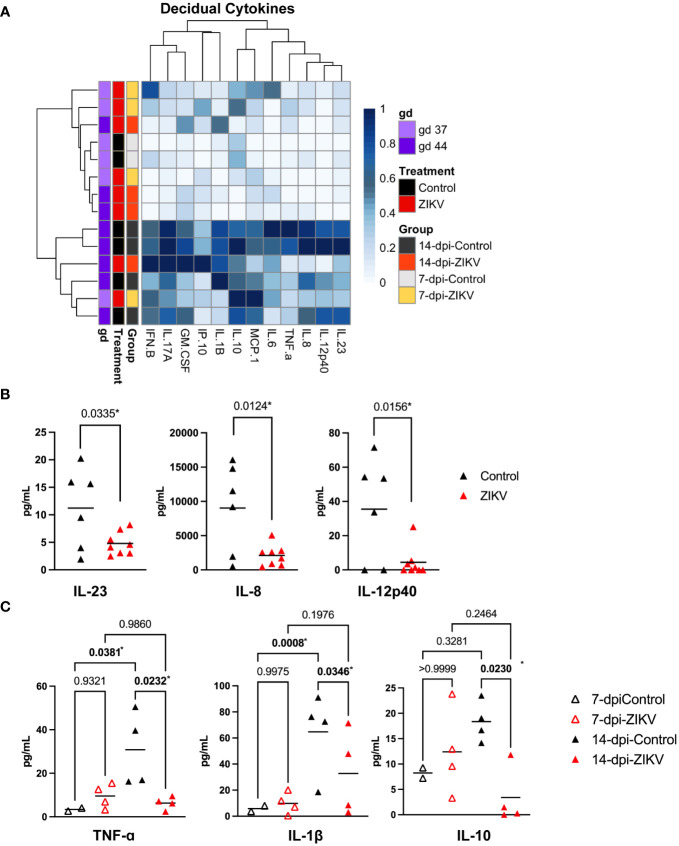
Cytokines and chemokines in decidual samples. **(A)** Heatmap of normalized cytokine and chemokine values (columns) from decidua samples (rows). Values are normalized by column. Samples are annotated on the left side of the heatmap, showing the treatment, group, and approximate gestational age in days (gd) of the sample. **(B)** Comparison of IL-23, IL-8, and IL-12p40 in decidua samples from ZIKV vs. controls. A t-test was used to determine statistical significance. **(C)** Comparisons of TNF-ɑ, IL-1β, and IL-10 at the group level. **(B, C)** P values for each comparison are shown. Significance was determined using a one-way ANOVA followed by followed by a Bonferroni’s comparison test. * indicates statistically significant p values.

Hierarchical clustering of normalized cytokines and chemokine values from decidua samples suggests that, overall, gestational age has the largest influence (**Figure 8A**). Cytokine levels were compared at both the treatment (ZIKV vs controls) and group (7-dpi-ZIKV, 14-dpi-ZIKV, 7-dpi-control, 14-dpi-control) to evaluate if ZIKV infection had an effect overall or only at specific time-points. Pro-inflammatory cytokines IL-23, IL-8, and IL-12p40 were found to be decreased in the decidua in pooled ZIKV samples compared to pooled controls (**Figure 8B**). When looking at the group level, additional pro-inflammatory cytokines TNF-ɑ and IL-1β were decreased in the 14-dpi-ZIKV samples compared to the 14-dpi-Controls, and the anti-inflammatory cytokine IL-10 was decreased in 14-dpi-ZIKV samples compared to the 14-dpi-Control samples (**Figure 8C**). Overall, cytokine and chemokine levels in decidual homogenates from ZIKV and control samples further support that the decidua does not have a pro-inflammatory response to ZIKV infection.

**Figure 9:**
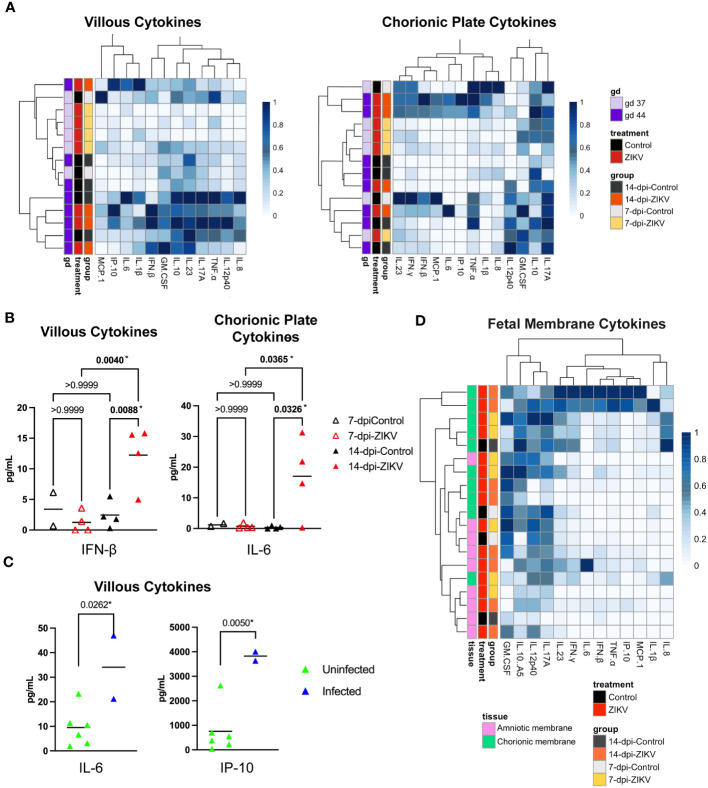
Cytokines and chemokines in placenta and fetal membranes. **(A)** Heatmaps of normalized cytokine and chemokine values (columns) from samples (rows). Chorionic villi samples are shown on the left panel and chorionic plate samples on the right. Values are normalized by column. Samples are annotated on the left side of the heatmaps, showing the treatment, group, and approximate gestational age in days (gd) of the sample. **(B)** Comparison of IFN-β levels in chorionic villous samples (left panel) and IL-6 values in chorionic plate samples (right panel) from ZIKV and controls. Significance was determined by a one-way ANOVA followed by a Bonferroni’s comparison test. **(C)** Comparison of IL-6 and IP-10 in chorionic villous samples between samples that were ZIKV-exposed but uninfected and samples with confirmed ZIKV infection in the villi. Significance was determined by a t-test. **(B, C)** P values for each comparison are shown; * indicates statistically significant p values. **(D)** Heatmap of normalized cytokine and chemokine values (columns) from samples (rows). Values are normalized by column. Samples are annotated on the left side of the heatmap, showing tissue (amniotic and chorionic membrane), the treatment, and the group.

Cytokine and chemokine levels were also assessed in the chorionic villi and chorionic plate of the placenta (**Figure 9A**). There was a significant increase in the level of IFN-β in the chorionic villi and a significant increase in the level of IL-6 in the chorionic plate in the 14-dpi-ZIKV samples compared to the 14-dpi-control samples (**Figure 9B**). Because infection in the chorionic villi was only seen in two out of eight ZIKV pregnancies (36), we compared the six ZIKV-exposed but uninfected chorionic villi with the two chorionic villi that were ZIKV-infected. We found that the ZIKV-infected chorionic villi had higher levels of IL-6 and IP-10 (**Figure 9C**). Overall, results suggest that in contrast to the decidua, ZIKV had a mild pro-inflammatory effect on the placenta.

The cytokine and chemokine levels in the chorionic and amniotic membranes were also evaluated (**Figure 9D**). Unfortunately, these samples were only collected from two control cases. Therefore, only the 7-dpi-ZIKV samples and 14-dpi-ZIKV were compared to each other, and no significant differences were found (**Supplementary Figure 6**).

## Discussion

The impact of maternal ZIKV infection on the immune environment at the maternal-fetal interface remains incompletely understood. In this study, we employed spectral flow cytometry and unbiased clustering to identify and phenotype decidual leukocytes in control and ZIKV-infected early pregnant rhesus macaques. First, our analysis found that decidual leukocytes represent a unique population that is distinct from that found in the PBMCs. We also found that T cells, not ILCs, were the predominant decidual leukocyte population. Finally, looking at the effect of ZIKV infection, we found no evidence to support our initial hypothesis that ZIKV infection results in a pro-inflammatory response in the decidua. On the contrary, we found that ZIKV infection was associated with mild phenotypic changes in decidual leukocytes that are consistent with an anti-inflammatory response.

### ZIKV infection was associated with mild anti-inflammatory changes in decidual leukocytes

*Ex vivo* studies using human decidual explants have shown that ZIKV infection results in pro-inflammatory responses (30, 31). Moström et al. evaluated the response to Asian-linage ZIKV infection *in vivo* in decidua samples from rhesus macaques 16–56 days post infection (44). However, the *in vivo* acute response to African-lineage ZIKV infection in the decidua remained unknown. Studies comparing the response to these ZIKV lineages using *in vitro*, *ex vivo*, and *in vivo* studies have found that infection with African-lineage ZIKV, in particular, generates a pro-inflammatory response (92–94). Most relevant to the current studies, Foo et al. found that macrophages collected from pregnant people respond to African-lineage ZIKV by undergoing pro-inflammatory changes (94). We, therefore, initially hypothesized that African-lineage ZIKV infection would result in a pro-inflammatory response in the decidua. However, in our evaluation of decidual leukocytes, we found little evidence to support our hypothesis.

In our evaluation of the cytotoxic T cells, helper T cells, ILCs, macrophages, DCs, and B cells in the decidua we found that ZIKV had the most pronounced effect on decidual macrophages. We found that a significantly higher proportion of decidual macrophages expressed CD163 in ZIKV-infected dams compared to controls. CD163 is a scavenger receptor that functions to phagocytose hemoglobin/haptoglobin, preventing oxidative damage and may play a role in the resolution of inflammation (57). CD163 expression has been shown to be induced by the anti-inflammatory cytokine IL-10 and suppressed by pro-inflammatory cytokines IFN-γ and TNF-ɑ and by LPS stimulation (95, 96). An increase in the expression of CD163 may be an example of the immune response co-opting an existing tolerance mechanism. Additional evidence of an attenuated macrophage response is the lack of change in the expression of CD86, an activation and costimulation marker expressed on pro-inflammatory macrophages commonly referred to as M1 (97). An increase in CD86 expression as well as HLA-DR expression is one way that macrophages can communicate with T cells to promote inflammation as part of a response to pathogens. Overall, phenotypic changes seen in decidual macrophages suggest an anti-inflammatory response.

The main finding within the ILCs was an increase in Eomes expression in the ZIKV samples. Eomes expression was found to be increased overall in the ILCs from ZIKV dams, and in our clustering of ILCs, we found that the increase in Eomes was seen in specific dNK Eomes+ clusters and in both ILC1 clusters. The exact role of Eomes in inflammation is currently unknown, however, there is evidence to suggest that Eomes may play a role in NK cell cytotoxicity (75). Although we found an increase in RORγt in ZIKV samples, we only saw this increase in less than 2% of the total ILCs, and thus it is not surprising that we did not see an increase in IL-17A levels in the decidua. Overall, we saw minimal evidence of phenotypic changes in the ILCs that are consistent with either a pro-inflammatory or anti-inflammatory response.

### *In vivo* decidual response differs from that observed in *ex vivo*

*Ex vivo* experiments using human decidual explants found that ZIKV infection caused an increase in several pro-inflammatory cytokines or the expression of their encoding mRNAs. Yet, in our evaluation of *in vivo* decidual cytokine levels, we only found a decrease in pro-inflammatory cytokines. There may be several explanations for the differences between *in vivo* and *ex vivo* results. Tissue explants may not accurately model how ZIKV infects and spreads through the decidua *in vivo*: while *in vivo* the virus must enter the tissue by crossing/infecting the maternal vasculature to subsequently infect decidual cells, when tissue explants are exposed to ZIKV *ex vivo*, decidual cells may be exposed directly to virus, bypassing the normal transendothelial route. This may result in a pro-inflammatory response that differs from that seen *in vivo*.

The lack of a pro-inflammatory response in the decidua may be a reflection of the focal nature of ZIKV infection in the decidua (36). As shown in Koenig et al., ZIKV infection in the decidua was limited to endovascular EVTs and small groups of infected macrophages (36). Within the ZIKV infected macrophages there may have been focal inflammatory responses by decidual leukocytes, but this inflammation was not extensive enough to be detected. Furthermore, the lack of an inflammatory response in the decidua to ZIKV infection may be the consequence of the relatively immune-privileged status of the decidua. Several mechanisms have been identified, including chemokine silencing in decidual stromal cells, to prevent aberrant inflammation in favor of maintaining a tolerant environment to support pregnancy (98–102). This possibility is supported by the fact that we do see an increase in IP-10 and IL-6 in the placental villi and an increase in IL-6 in the chorionic plate but no increase in any pro-inflammatory cytokines in the decidua associated with ZIKV infection. Our finding that ZIKV infection does not cause pro-inflammatory changes in the decidua is consistent with our histopathological findings reported in Koenig et al. (36). We found that ZIKV had no substantial effect at 7 and 14 dpi on decidual histopathology. Somewhat surprisingly, we did not see an increase in inflammatory cytokines in the chorionic and amniotic membranes between the 7-dpi-ZIKV and 14-dpi-ZIKV, given that we have previously shown these cells to be significantly infected by ZIKV (36). Unfortunately, we were not able to collect chorionic and amniotic membrane samples from more than two of the control pregnancies.

### T cells were the dominant decidual leukocyte

Our finding that T cells, not ILCs, were the dominant decidual leukocyte is consistent with the study published by Mostrom et al. (44). However, our results are inconsistent with what has been previously published by our group, who found ILCs, specifically decidual NK cells, to be dominant (43, 51). The discrepancies between this current study and prior work are likely a combination of different isolation methods and the application of modern flow cytometry techniques. Decidual leukocyte isolation methods have been shown to influence the number of cells obtained, cell viability, and marker expression (103, 104).

The Slukvin et al study, Dambaeva et al study, and this current study all isolated decidual leukocytes using a combination of mechanical and enzymatic digestion (43, 51). The Slukvin and Dambaeva studies used enzymes and shaker flask to gently disperse the tissue over approximately an hour and a half; while this current study used a MACS dissociator and a lower concentration of similar enzymes to digest the decidua over a shorter period of time (43, 51). To fully evaluate the discrepancies seen in the frequency of T cells vs. ILCs in the decidua, leukocytes could be profiled in histological sections, preserving the spatial context of the leukocytes. Decidual leukocytes have been evaluated in rhesus macaque decidual tissue sections using immunohistochemistry (105, 106). However, staining techniques that allow for the simultaneous detection of several linage markers would be needed to distinguish NK cells, T cells, and antigen presenting cells. Given the substantial technical development that this would involve, this type of analysis is beyond the scope of this study. Ultimately, further studies are needed to thoroughly evaluate the discrepancies in the frequencies seen in NK and T cells isolated from rhesus macaques in early pregnancy.

In conclusion, this study successfully applied high-dimensional spectral flow cytometry to identify and phenotype decidual leukocytes. Major leukocyte populations were identified within the decidua and the PBMCs. We found that ZIKV infection was overall associated with mild phenotypic changes in decidual leukocytes that were consistent with an anti-inflammatory response. The cytokine and chemokine analysis found further evidence of an anti-inflammatory decidual response. Evaluation of cytokines and chemokines in other tissues of the conceptus found an increase in inflammatory cytokines in placental samples that had infection in the mesenchymal core of the chorionic villi and in the chorionic plate, suggesting that ZIKV is capable of inducing a placental pro-inflammatory response. However, this was not seen within the decidua. We believe that the lack of a pro-inflammatory response in the decidua to ZIKV infection is consistent with the results presented in Koenig et al. (36), showing that ZIKV infection in the decidua is mainly restricted to endovascular EVTs and that ZIKV infection causes no clear pathology in the decidua. Our findings emphasize the immunological state of the gravid uterus as a relatively immune privilege site that prioritizes tolerance of the fetus over mounting a pro-inflammatory response to clear infection.

## Data availability statement

The datasets presented in this study can be found in online repositories. The names of the repository/repositories and accession number(s) can be found below: http://flowrepository.org/, FR-FCM-Z6UU;FR-FCM-Z6VZ;FR-FCM-Z6V3.

## Ethics statement

The animal study was approved by University of Wisconsin Institutional Animal Care and Use Committee. The study was conducted in accordance with the local legislation and institutional requirements.

## Author contributions

MK: Conceptualization, Formal analysis, Investigation, Methodology, Visualization, Writing – original draft, Writing – review & editing. JV: Investigation, Methodology, Writing – review & editing. FJ: Investigation, Writing – review & editing. AM: Project administration, Writing – review & editing. AS: Conceptualization, Supervision, Writing – review & editing. TG: Conceptualization, Funding acquisition, Project administration, Resources, Supervision, Writing – review & editing.
