# Supplementary material for: Decidual leukocytes respond to African lineage Zika virus infection with mild anti-inflammatory changes during acute infection in rhesus macaques
Source: Front Immunol. 2024 Mar 7;15:1363169. doi: 10.3389/fimmu.2024.1363169 (PMC10954895; doi:10.3389/fimmu.2024.1363169)
Supplement: Supplementary file 1 [file DataSheet_1.pdf]

Supplementary Material

Supplementary Material

1 Supplementary Figures and Tables

1.1 Supplementary Figures

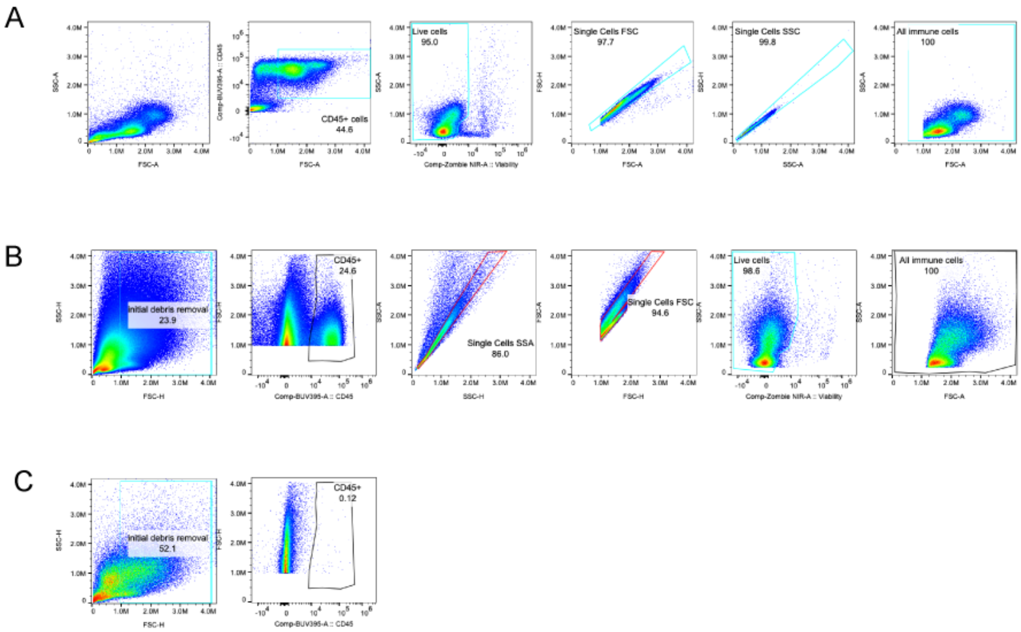

Supplemental Figure 1. Initial gating of PBMCs and Decidual cells. Gating scheme for “all immune cells” in representative PBMC **(A)** and decidua **(B)** samples. **(A)** PBMCs were gated selecting CD45+ cells that were larger than the debris shown on the FSC-A axis. Cells were further gated to select live, single cells resulting in a population of “all immune cells”. Samples with more than 50,000 “all immune cells” were downsampled to 50,000. **(B)** Gating scheme for decidual cells. Using the FSC-A of the PBMC samples as a guide, decidual samples were first gated to remove debris. Leukocytes were then selected based on their expression of CD45. Cells were further gated to select live, single cells of “all immune cells”. **(C)** Shows a fluorescence-minus-one (FMO) control-stained decidua sample that is labeled with all markers but CD45 that was used to guide the gating of CD45+ decidual cells. **(A)(B)** Samples shown are paired samples belonging to one dam.

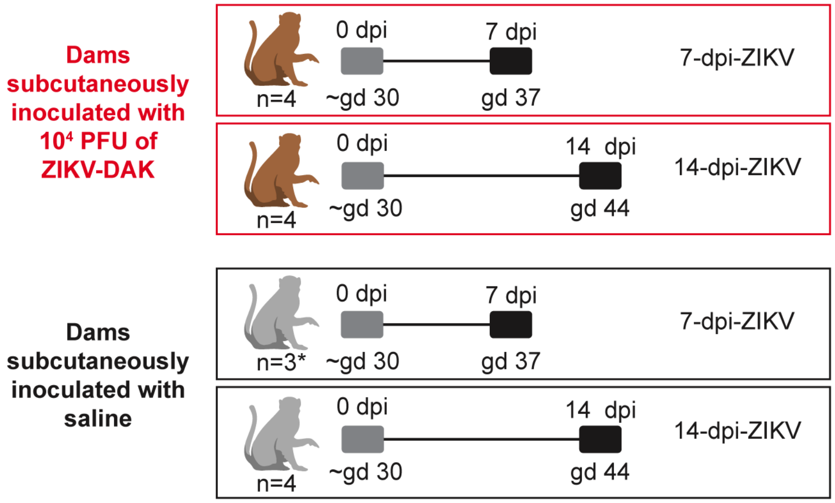

Supplemental Figure 2. Study overview. Eight dams were subcutaneously inoculated with 104 plaque forming units (PFU) of a This study was done with four distinct groups of dams. Eight pregnant female rhesus macaques were subcutaneously inoculated with 10^4 plaque-forming units (PFU) of a Senegal isolate of African-lineage Zika virus ZIKV/*Aedes africanus*/SEN/DAK-AR-41524/1984 (ZIKV-DAK), at approximately gestational day (gd) 30. Four of these dams had their pregnancies surgically terminated seven days post-infection (dpi), and four had their pregnancies surgically terminated at 14 dpi (7-dpi-ZIKV and 14-dpi-ZIKV). Seven additional pregnant female rhesus macaques were injected with sterile saline and subject to the same experimental sampling regimen as the ZIKV-infected pregnancies. Four control pregnancies were surgically terminated seven days post-saline-injection and four at 14 days post-saline-injection. These two control groups are referred to in the manuscript as 7-dpi-control and 14-dpi-control. *One of the control dams was randomly assigned to the control seven dpi group twice; all statistical analyses have been done with an average of the results from her two pregnancies.

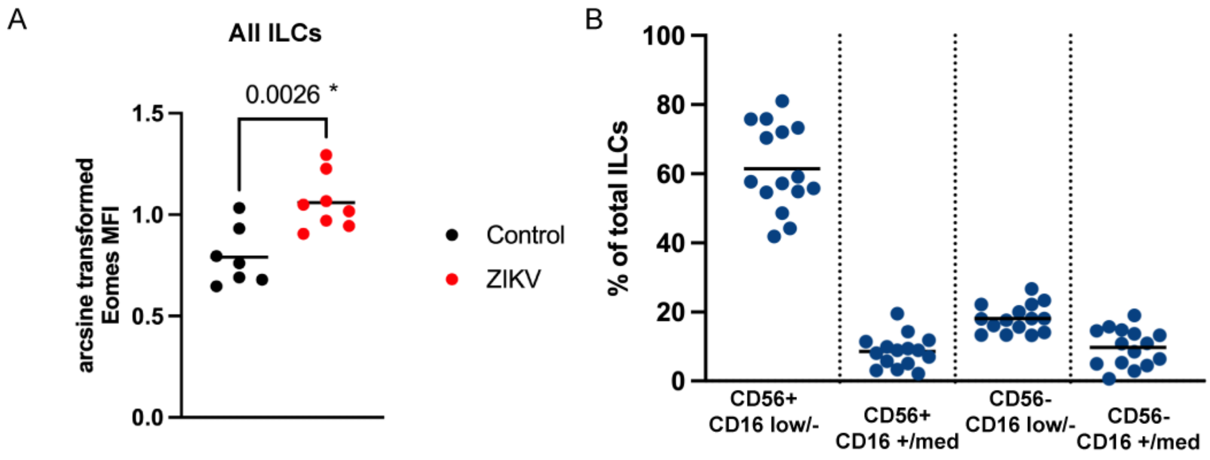

Supplemental Figure 3. Additional ILC data. (A) Scatter plot showing the arcsine transformed MFI of Eomes in Control and ZIKV ILC samples. **(B)** Scatter plot showing the frequency of different ILCs populations based on their expression of CD56 and CD16 from all samples.

A

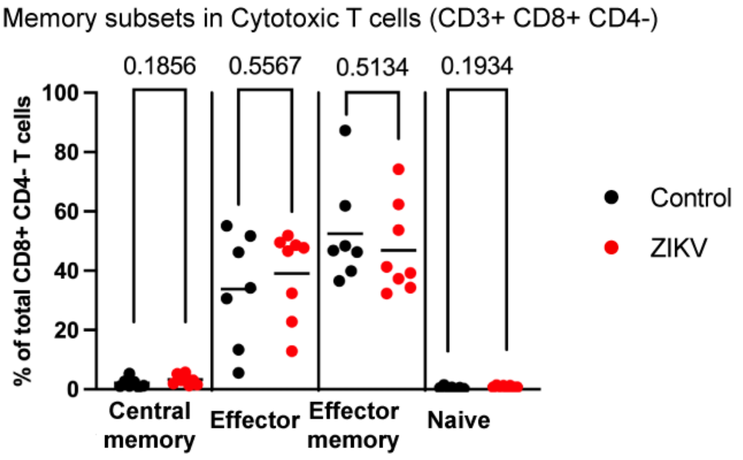

B

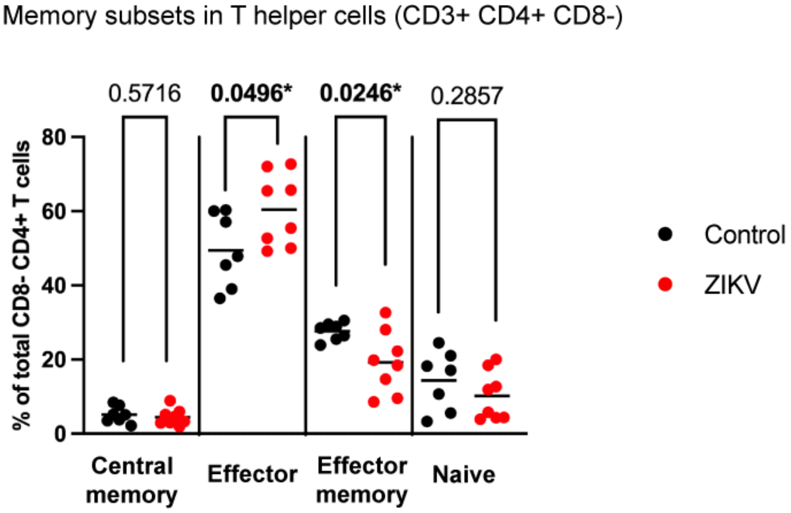

Supplemental Figure 4. Memory subsets in cytotoxic and helper T cells in the decidua. Traditional gating of memory subtypes of (A) cytotoxic T cells (CD3+ CD8+ CD4-) and (B) T helper (CD3+ CD4+ CD8-) cells. **(A)** Central memory T cells were defined by CD45RA-, CCR7+, effector as CD45RA^{high+}, CCR7-, effector memory as CD45RA^{med+}, CCR7-, and naive as CD45RA^{high+}, CCR7+. **(B)** Central memory T cells were defined by CD45RA-, CCR7+, effector as CD45R+, CCR7-, effector memory as CD45RA-, CCR7-, and naive as CD45RA+, CCR7+. P values are shown, *indicates a significant p value.

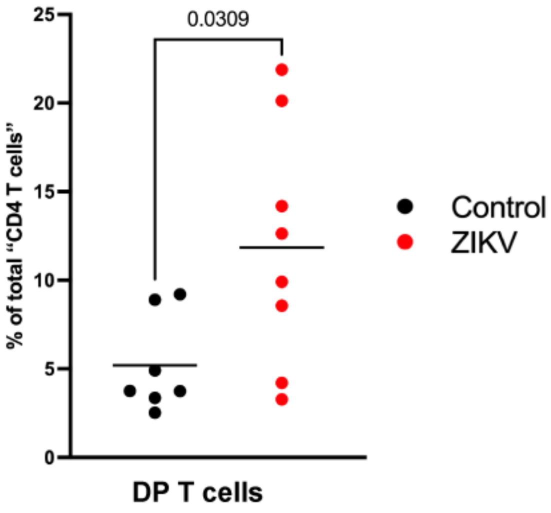

Supplemental Figure 5. Frequency of DP T cells in total "CD4 T cells". Scatter plot showing frequency of DP T cells in total "CD4 T cells" in pooled ZIKV and control.

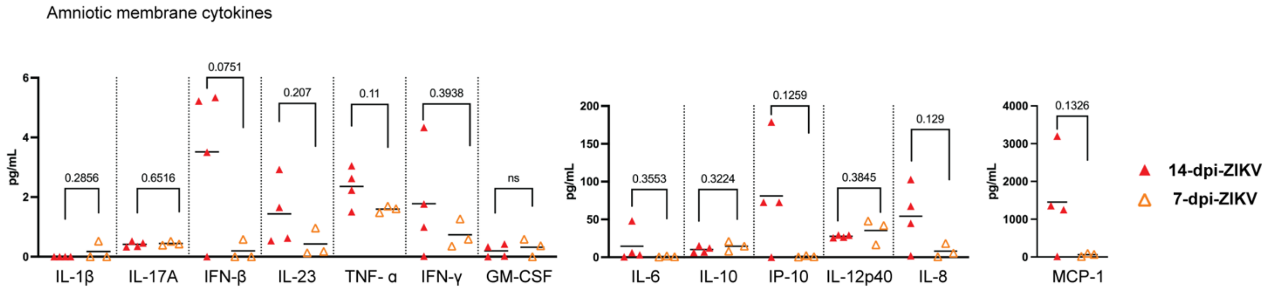

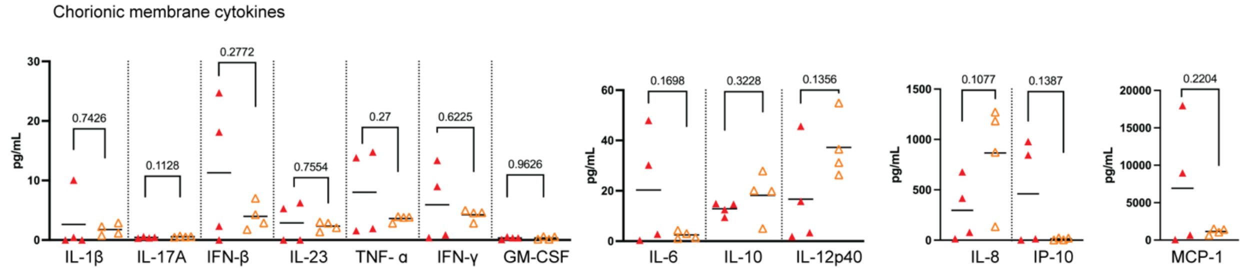

Supplemental Figure 6. Cytokines levels in chorionic and amniotic membrane from ZIKV infected pregnancies. Statistical comparisons were made using a t-test, p-values are shown.

1.2 Supplementary Tables

Supplementary Table 1. Pregnancy and sample details.

Sample	Treatment	Group	treatment gestational age (days)	days post infection of pregnancy termination	pregnancy termination gestational age (days)	day of PBMC sample used in relation to decidua collection (0 = same day)	Sex of Fetus
**07-1**	ZIKV	7-dpi-ZIKV	30	7	37	0	**Female**
**07-2**	ZIKV	7-dpi-ZIKV	29	7	36	0	**Female**
**07-3**	ZIKV	7-dpi-ZIKV	30	7	37	0	**Female**
**07-4**	ZIKV	7-dpi-ZIKV	30	7	37	0	**Female**
**14-1**	ZIKV	14-dpi-ZIKV	29	14	43	0	**Male**
**14-2**	ZIKV	14-dpi-ZIKV	28	14	42	0	**Male**
**14-3**	ZIKV	14-dpi-ZIKV	29	14	43	0	**Male**
**14-4**	ZIKV	14-dpi-ZIKV	32	14	46	0	**Female**
**30/14-C1**	Control	14-dpi-Control	30	14	44	0	**Female**
**30/14-C2**	Control	14-dpi-Control	30	15	45	- 7	**Female**
**30/14-C3**	Control	14-dpi-Control	31	14	45	- 3	**NA**
**30/14-C4**	Control	14-dpi-Control	30	14	44	0	**Female**
**30/7-C1**	Control	7-dpi-Control	30	7	37	- 4	**Male**
**30/7-C1B**	Control	7-dpi-Control	30	7	37	0	**Male**
**30/7-C2**	Control	7-dpi-Control	30	7	37	0	**Female**
**30/7-C3**	Control	7-dpi-Control	32	7	38	0	**Male**
**NA = Not determined**							

Supplemental Table 2. Antibody and flow reagent details.

Marker	Fluor	peak channel	Clone	Company	cat #	lot#	Stock concentration	amount used in 50ul test (ul)
CD45	BUV395	UV02	D058-1283	BD Biosciences	564099	1198879	0.075 mg/ml	0.665
CCR6	BUV496	UV07	11A9	BD Biosciences	612948	2047182	200 ug/mL	1.25
CD45RA	BUV563	UV09	5H9	BD Biosciences	741411	1187899	0.2 mg/mL	0.6
CD4	BUV615	UV10	SK3	BD Biosciences	612987	1078219	0.125 ug/test	1
CD69	BUV661	UV11	FN50	BD Biosciences	750213	2208987	0.2 mg/mL	1.25
CD8a	BUV805	UV16	SK1	BD Biosciences	612889	1040596	0.5 ug /5 ul	0.8
FoxP3	BV421	V01	206D	Biolegend	320123	B275759	100 ug/mL	0.5
CD16	BV480	V05	3G8	BD Biosciences	566108	12375229	400 ug/mL	1.25
CD14	BV570	V08	M5E2	Biolegend	301832	B312501	150 ug/mL	1.25
CD49a	BV605	V10	SR84	BD Biosciences	742359	283958	0.2 mg/mL	1.25
CD163	BV650	V11	GHI/61	BD Biosciences	563888	299243	NA	0.3125
CD3	BV711	V13	SP34-2	BD Biosciences	740807	2208984	0.2 mg/ml	2.5
CCR7	BV785	V15	G043H7	Biolegend	353230	B340912	200 ug/mL	1.25
CD56	BB515	B01	B159	BD Horizon	564488	2045872	0.2 mg/mL	1.25
CD20	PerCP Cy5.5	B09	2H7	Biolegend	302326	B28496	50 ug/mL	2
RORy-t	PE	YG01	AFKJ5-9	Invitrogen	12-6988-82	2158265	0.2 mg/mL	0.5
CD11c	PE/Dazzle 594	YG03	3.9	Biolegend	301642	B311535	100 ug/mL	1.25
T-bet	PE-Cy5	YG05	ebio4B10	Invitrogen	12-6988-82	2313112	0.2 mg/mL	0.5
CD127	PE/Fire700	YG07	A019D5	Biolegend	351366	B322711	50 ug/mL	1.25
NKp46	PE-Cy7	YG09	-	Beckman Coulter	B38703	200035	50 ug/mL	2.5
CD86	PE/Fire810	YG10	IT2.2	Biolegend	B343059	3343059	200 ug/mL	2
*DC-SIGN	APC	R01	DCN46	BD Biosciences	551545	9123890	NA	1.25
Eomes	eFluor 660	R02	WD1928	Invitrogen	50-4877-42	2106865	0.03ug/5ul	0.5
Viability	Zombie NIR	R06	NA	Biolegend	423105	B334686		1:4000 dilution
HLA-DR	APC/Fire810	R08	L243	Biolegend	307674	B342759		1.25

TrueStain Fc block	Biolegend			2.5 ul/ per 50ul reaction
Monocyte Block	Biolegend	426101	B333954	2.5 ul/ per 50ul reaction
Brilliant Stain Buffer	BD Biosciences	563794	8330948	volume used = (50ul - (volume of surface antibodies + volume of blocks))

*DC-SIGN antibody was included in the staining cocktail, but was excluded from analysis due to poor signal

Supplementary table 3. Macrophage marker expression.

**Sample**	**Treatment**	**% of Macs CD56+**	**% of Macs CD16+**	**% of Macs CD163+**	**% of Macs CD11c+**	**% of Macs CD86+**	**% of Macs CD69+**
07-2	ZIKV	86	42.7	84.3	75.6	66.8	32.6
14-2	ZIKV	92	23	78.8	77.7	63.3	28.2
14-1	ZIKV	97.1	66.7	85.6	76.4	75.3	34.2
14-3	ZIKV	97	46.7	84.5	56.6	77.9	37.3
07-4	ZIKV	84.4	61.1	89	70.7	64	51.4
14-4	ZIKV	92.2	34.6	89.6	87.3	90	56.2
07-1	ZIKV	94.1	24.4	83.9	62.2	71.7	31.7
07-3	ZIKV	89.8	65.1	82.5	90.5	85.7	20.3
30/14-C3	Control	98.1	23.3	73.2	78.2	73.1	15.5
30/14-C2	Control	95.8	21.6	80	57.1	63.3	15.4
30/14-C1	Control	94.3	44.4	76	95.1	79.8	11.3
30/7-C3	Control	97.1	15.1	63	94.8	77.2	31.1
30/7-C2	Control	93.3	33.5	79.7	72.9	64.5	9.91
30/7-C1	Control	96.6	36.1	80.95	84.9	70.95	24.2
30/14-C4	Control	92.3	38.7	77.5	84.2	66.1	22.9
average of all		93.34	38.47	80.57	77.61	72.64	28.15
average of control		95.36	30.39	75.76	81.03	70.71	18.62
average of ZIKV		91.58	45.54	84.78	74.63	74.34	36.49
standard deviation of all		4.08	16.22	6.65	12.34	8.34	13.45
